# GSTT1 Null Genotype Contributes to Lung Cancer Risk in Asian Populations: A Meta-Analysis of 23 Studies

**DOI:** 10.1371/journal.pone.0062181

**Published:** 2013-04-24

**Authors:** Xin Yang, Man-Tang Qiu, Jing-Wen Hu, Xiao-xiao Wang, Feng Jiang, Rong Yin, Lin Xu

**Affiliations:** 1 The First Clinical College of Nanjing Medical University, Nanjing, China; 2 Department of Thoracic Surgery, Nanjing Medical University Affiliated Cancer Hospital, Cancer Institute of Jiangsu Province, Nanjing, China; 3 Department of Bio-statistics, Georgia Health Science University, Augusta, Georgia, United States of America; 4 The Fourth Clinical College of Nanjing Medical University, Nanjing, China; Baylor College of Medicine, United States of America

## Abstract

**Background:**

Genetic variation in glutathione S-transferases (GSTs) may contribute to lung cancer risk. Many studies have investigated the correlation between the Glutathione S-transferase T1 (GSTT1) null genotype and lung cancer risk in Asian population but yielded inconclusive results.

**Methodology/Principal Findings:**

We performed a meta-analysis of 23 studies including 4065 cases and 5390 controls. We assessed the strength of the association of GSTT1 with lung cancer risk and performed sub-group analyses by source of controls, smoking status, histological types, and sample size. A statistically significant correlation between GSTT1 null genotype and lung cancer in Asian population was observed (OR = 1.28, 95% CI = 1.10, 1.49; P_heterogeneity_<0.001 and I^2^ = 62.0%). Sub-group analysis revealed there was a statistically increased lung cancer risk in ever-smokers who carried the GSTT1 null genotype (OR = 1.94, 95% CI = 1.27, 2.96; P heterogeneity = 0.02 and I^2^ = 58.1%). It was also indicated that GSTT1 null genotype could increase lung cancer risk among population-based studies (OR = 1.25, 95% CI = 1.04, 1.50; P_heterogeneity_ = 0.003 and I^2^ = 56.8%). The positive association was also found in studies of sample size (≤500 participants) (OR = 1.34, 95% CI = 1.10, 1.62; P_heterogeneity_<0.001 and I^2^ = 65.4%).

**Conclusions:**

These meta-analysis results suggest that GSTT1 null genotype is associated with a significantly increased risk of lung cancer in Asian population.

## Introduction

Lung cancer is the most common malignancy worldwide. It is a leading cause of cancer death in men and women in the United States [1]. In recent years, the incidence of lung cancer in Asia increased rapidly. It has become one of the greatest threats to human health. Tobacco smoking, family history and susceptible gene mutations are main risk factors of lung cancer. Recently, various gene polymorphisms contributing to lung cancer risk have been discovered, such as DNA repair genes family (XRCC1, hOGG1, XPD, XPA, XRCC3) [2], [3], [4], [5], [6], cytochrome P450 (CYP450) [7], glutathione S-transferases family (GSTs) [8], and MicroRNAs (miRNAs) [9], [10], [11].

GSTs contain a variety of function isozymes, which are involved in the metabolic detoxification of reactive electrophilic compounds [12]. The glutathione S-transferases (GSTs) family contains six members in human: GSTA (α), GSTM (μ), GSTP (π), GSTS (σ), and GSTT (θ) [13], [14]. Glutathione S-transferase T1 (GSTT1) belongs to GSTT (θ), which has been identified in human liver. It is located on 22q11.23, and encodes a protein consisting of 240 amino acids. The length of GSTT1 gene is 8092bp with 5 exons and 4 introns [15]. The genotype of GSTT1 allele homozygous deletion is GSTT1 null. In Asian population, the frequency of GSTT1 null genotype is higher compared with other population [16]. Recently, there are a number of published studies focusing on the relationship between GSTT1 null genotype and lung cancer risk. In 1996, Deakin first reported that GSTT1 null genotype was associated with an increased susceptibility to total ulcerative colitis, but was not increased in the lung, oral or gastric cancer cases [17]. Thereafter, a lot of studies have been carried out and yielded different or even controversial results. For example, some studies found that GSTT1 null genotype was associated with an increased risk for lung cancer in Asian population [18], [19], while other studies reported negative results [20], [21].

To determine the correlation between GSTT1 null genotype and lung cancer risk in Asian population, we performed this meta-analysis by summarizing reported case–control studies, calculating the estimate of overall lung cancer risk and evaluating influence of smoking status and histological types.

## Methods

### Literature Search Strategy

Eligible case-control studies included in our analysis were extracted by electronic search of databases (PubMed, EMBASE) and manual search of references of relative articles and reviews. Search terms were keywords relating to the GSTT1 gene (e.g., “Glutathione S-transferaseT1”, and “GSTT1”) in combination with words related to lung cancer (e.g., “lung”, combined with “cancer”, “carcinoma”, “tumor” or “neoplasms”) and polymorphism or variation. The last research was performed on January 6, 2013. All relevant reports identified were included with no restriction.

### Inclusion and Exclusion Criteria

The major inclusion criteria were (a) case–control studies or cohort studies; (b) investigating the association between GSTT1 null genotype and lung cancer risk; (c) Asian population; (d) available genotype distribution information in cases and controls or odds ratio (OR) with its 95% confidence intervals (CIs). The major reasons for exclusion of studies were (a) reviews and repeated literatures; (b) case-only studies; (c) studies without detail genotype frequencies.

### Data Extraction

Data of eligible studies were extracted independently by two investigators and the following data were collected: name of first author, year of publication, country where the study was conducted, age, sex, histological types, source of control, number of cases and controls, genotype frequency in cases and controls. Histological types were classified as squamous cell carcinoma (SqCC), adenocarcinoma (AC), small cell carcinoma (SCC) and others. All eligible studies were defined as hospital-based (HB) or population-based (PB) according to the control source. The two investigators directly extracted genotype frequency or estimated odds ratio of the papers. Discrepancies were discussed among all authors until they reached consensus on each item.

### Statistical Analysis

ORs with 95% CIs were used to measure the strength of association of GSTT1 null genotype with lung cancer risk. The pooled ORs of GSTT1 null genotype mentioned above was compared with GSTT1 present genotype between cases and controls. A 95% CI was used for statistical significance test and a 95% CI without 1 for OR indicating a significant increased or reduced cancer risk. Sensitivity analyses were performed to identify individual study’ effect on pooled results and test the reliability of results. The fixed effects model (Mantel-Haenszel method) was used when there was no significant heterogeneity; otherwise, the random-effects model (the Der Simonian and Laird method) was utilized [22]. Chi-square based Q test was used to check the statistical heterogeneity between studies, and the heterogeneity was considered significant when p<0.10[23]. The quantity I^2^ presented variation in OR attributable to heterogeneity [24]. Stratification and meta-regression analyses were performed to explore the potential source of heterogeneity among studies. Publication bias was detected with Begg’s funnel plot [25] and the Egger’s linear regression test [26], and p<0.05 was considered significant. All P values are two-sided. Statistical analyses were done with Stata (version 12.1; Stata Corp, College Station, Texas USA).

## Results

### Characteristics of Studies

**Figure 1:**
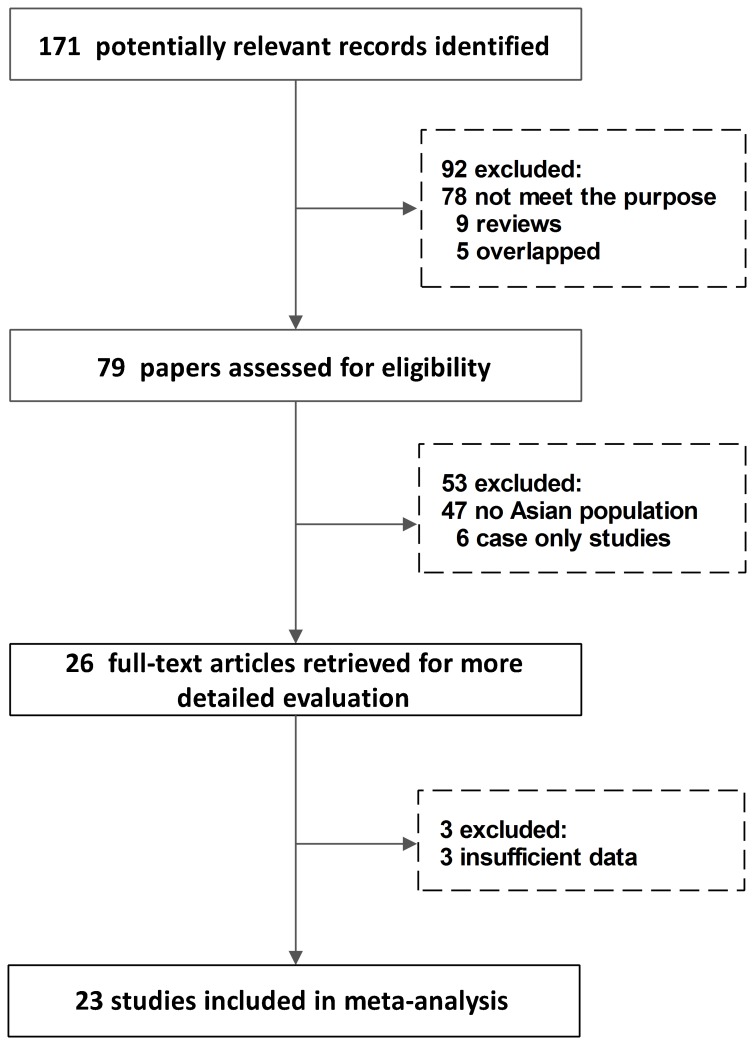
The flow chart of the included studies for a meta-analysis of GSTT1 null genotype and lung cancer risk in Asian population.

As shown in Figure 1, a total of 23 studies [18]–[21], [27]–[45] were identified according to inclusion and exclusion criteria. The 23 studies included 4065 cases and 5390 controls. The detailed characteristics of the eligible studies included in this meta-analysis are shown in Table 1.

**Table 1:** Characteristics of Eligible Studies.

First Author	Year	Country	Histological types	Source of controls	Mean age of case/control	Male (%) of case/control	Smoke(%) ofcase/control	NO. of cases	NO. of controls
**Lan**	2000	China	NA	PB	55/55	64.8/64.8	100/100	122	122
**London**	2000	China	NA	PB	NA/NA	NA/NA	NA/NA	232	710
**Kiyohara**	2000	Japan	SqCC = 27.9%, SCLC = 14.0%, AC = 46.5%, LCC = 4.7%, others = 7.0%	PB	63.8/59	100/100	45.5/68.6	86	88
**Zhao**	2001	Singapore	NA	HB	65.5/63.6	0/0	41.2/9.6	233	187
**Sunaga**	2002	Japan	AC = 100%	HB	63/65	62.6/71.7	60.6/63.8	198	152
**Wang**	2003	China	AC = 100%	PB	56.5/54.5	64.3/66.4	42.9/66.4	112	119
**Liang**	2004	China	SqCC = 41.4%, AC = 58.6%	HB	60.5/60.5	70.4/70.4	NA/NA	152	152
**Sobti**	2004	India	SqCC = 71.0%, SCLC = 24.0%, AC = 4.0%, LCC = 1.0%	PB	55.5/50.9	95/96.1	86.0/77.6	100	76
**Chan**	2004	China	SqCC = 16.6%, AC = 55.5%, others = 27.9%	PB	53.8/49.3	67.2/59.4	56.8/40.1	229	197
**Yuan**	2005	China	SqCC = 46.7%, AC = 40.7%, others = 12.7%	PB	57.1/54.7	76.7/77	65.3/34.2	150	152
**Sreeja**	2005	India	NA	PB	58.2/56.1	91.1/87.7	59.9/45.2	146	146
**Chen**	2006	China	SqCC = 52.6%, AC = 44.3%, others = 3.1%	PB	56.6/55.8	43.3/48.7	0/0	97	197
**Lee**	2006	Korea	SqCC = 43.3%, SCLC = 18.1%, AC = 28.1%, others = 10.5%	HB	61/62.1	100/100	91.7/81.4	171	196
**Yang**	2007	Korea	SqCC = 22.3%, SCLC = 9.7%, AC = 54.7%, others = 13.3%	PB	55.4/48.3	67.6/61.8	62.3/50.1	318	353
**Sobti**	2008	India	SqCC = 63.5%, SCLC = 15.8%, AC = 7.2%, others = 7.9%	HB	56.9/56.4	86/81.4	81.4/74.2	151	151
**Sreeja**	2008	India	NSCLC = 81.0%, SCLC = 9.5%, others = 9.5%	HB	57.8/56.2	86.3/87.2	68.2/56.4	211	211
**Klinchid**	2009	Thailand	SqCC = 39.6% AC = 60.4%	PB	59.8/57.8	64/46	80.0/34.0	91	82
**Kumar**	2009	India	SqCC = 23.0%, SCLC = 14.0%, AC = 38.0%, LCC = 6.0%, others = 20.0%	PB	42.6/39.8	87.1/80.2	81.0/100	93	253
**Yadav**	2010	India	NA	PB	58/58	76.2/68.3	80.0/47.0	101	221
**Tamaki**	2011	Japan	SqCC = 24.5%, SCLC = 21.4%, AC = 41.7%, LCC = 0.5%, others = 11.9%	HB	80.3/80.3	67.2/69	70.3/51.7	192	203
**Fowke**	2011	China	NA	PB	NA/NA	0/0	NA/NA	209	787
**Kiyohara**	2012	Japan	SqCC = 28.4%, SCLC = 14.9%, AC = 52.4%, LCC = 4.3%	HB	66.1/55.9	62.1/74.7	66.9/44.8	462	379
**Wang**	2012	China	SqCC = 49.3%, SCLC = 13.4%, AC = 33.0%, others = 4.3%	PB	61.4/57.4	69.4/63.7	NA/NA	209	256

SqCC: squamous cell carcinoma; AC: adenocarcinoma; SCLC: small cell carcinoma; LCC: large cell carcinoma; NSCLC: non–small cell carcinoma; PB: population-based; HB: hospital-based.

### Meta-analysis Results

**Figure 2:**
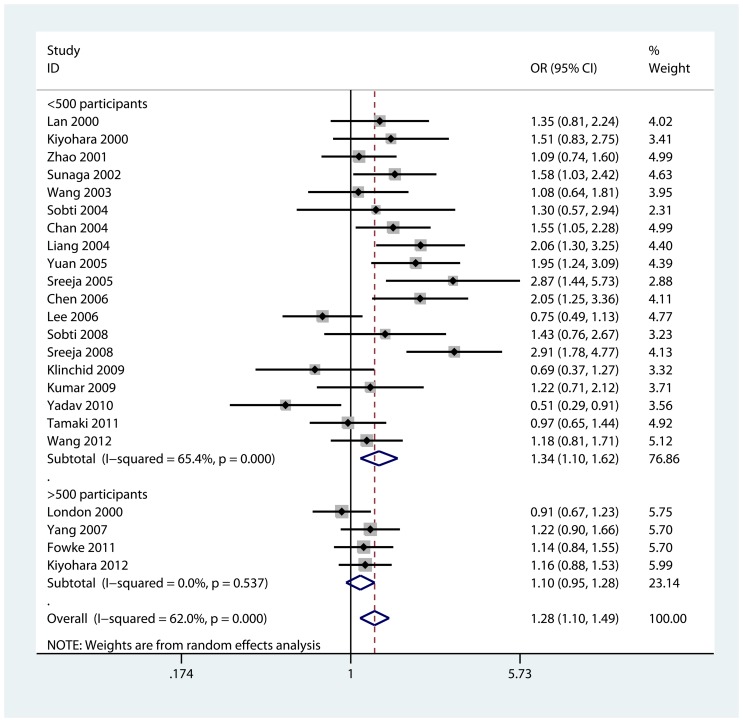
Forest plot for the association between GSTT1 null genotype and lung cancer risk in Asian population on stratification by sample size.

**Figure 3:**
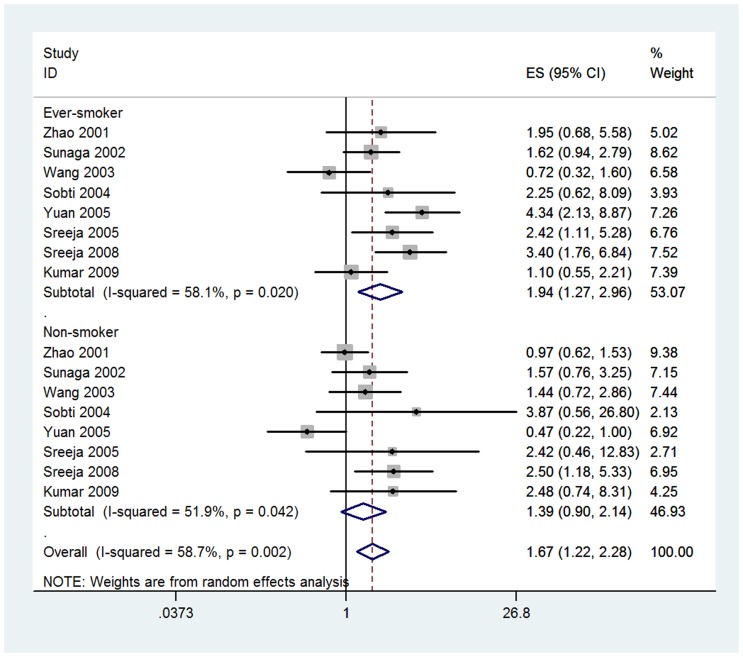
Forest plot for the association between GSTT1 null genotype and lung cancer risk in Asian population on stratification by smoking status.

We observed a statistically significant correlation between GSTT1 null genotype and lung cancer in Asian population (OR = 1.28, 95% CI = 1.10, 1.49; P_heterogeneity_<0.001 and I^2^ = 62.0%, Figure 2). As shown in Table 2, we performed sub-group analyses to investigate the effects of source of controls, smoking status, histological types, and number of participants. We first calculated the pooled OR for the risk of GSTT1 null genotype stratified by source of controls. There was a statistically significant link between GSTT1 null genotype and lung cancer in population-based studies (OR = 1.25, 95% CI = 1.04, 1.50; P_heterogeneity_ = 0.003 and I^2^ = 56.8%) but not in hospital-based studies (OR = 1.20, 95% CI = 0.95, 1.52; P_heterogeneity_ = 0.001 and I^2^ = 72.4%) (Figure S1). When stratified by smoking status, there was a statistically increased lung cancer risk in ever-smokers (OR = 1.94, 95% CI = 1.27, 2.96; P_heterogeneity_ = 0.02 and I^2^ = 58.1%), while we did not found any significant association in non-smokers (OR = 1.39, 95% CI = 0.90, 2.14; P_heterogeneity_ = 0.042 and I^2^ = 51.9%, Figure 3). In the sub-group analyses of “squamous cell carcinoma”, “adenocarcinoma”, and “small cell carcinoma”, we did not found any significant correlation between GSTT1 null genotype and specific histological type of lung cancer risk (Figure S2). When restricting the analysis to number of participants, GSTT1 null genotype was significantly associated with lung cancer risk in studies of sample size (≤500 participants), (OR = 1.34, 95% CI = 1.10, 1.62; P_heterogeneity_<0.001 and I^2^ = 65.4%) but not in studies of sample size (>500 participants) (OR = 1.10, 95% CI = 0.95, 1.28; P_heterogeneity_ = 0.537 and I^2^ = 0.0%, Figure 2).

**Table 2:** Meta-analysis Results.

Characteristics	N	Case/control	Heterogeneity test		OR(95% CI)	P_Egger’s test_	P_Begg’s test_
			I^2^	P			
**Total**	23	4065/5390	62.0%	<0.001	1.28(1.10,1.49)	0.115	0.205
**Source of controls**							
**Population**	15	2295/3759	56.8%	0.003	1.25(1.04,1.50)	0.262	0.428
**Hospital**	7	1559/1420	72.4%	0.001	1.20(0.95,1.52)	0.456	0.548
**Smoking status**							
**Ever-smoker**	8	792/579	58.1%	0.02	1.94(1.27,2.96)	0.972	0.902
**Non-smoker**	8	448/711	51.9%	0.042	1.39(0.90,2.14)	0.186	0.174
**Histological types**							
**SqCC**	7	395/617	69.8%	0.003	1.37(0.85,2.20)	0.524	0.548
**AC**	9	525/893	66.1%	0.003	1.26(0.89,1.78)	0.616	0.466
**SCLC**	3	138/285	0.0%	0.644	1.05(0.57,1.95)	0.239	0.296
**Sample size**							
**≤500 participants**	19	2844/3161	65.4%	<0.001	1.34(1.10,1.62)	0.483	0.726
**>500 participants**	4	1221/2229	0.0%	0.537	1.10(0.95,1.28)	0.86	1

SqCC: squamous cell carcinoma; AC: adenocarcinoma; SCLC: small cell carcinoma; P: p value for heterogeneity;

### Evaluation of Heterogeneity

Table 2 showed the heterogeneity of studies in each comparison. In order to investigate the source of heterogeneity, we explored variables as source of controls, smoking status, histological types and sample size with meta-regression. Meta-regression results revealed that histological types (p = 0.33), sample size (p = 0.26), source of control (p = 0.20), and smoking status (p = 0.38) did not contribute to the source of heterogeneity.

### Sensitivity Analyses and Publication Bias

**Figure 4:**
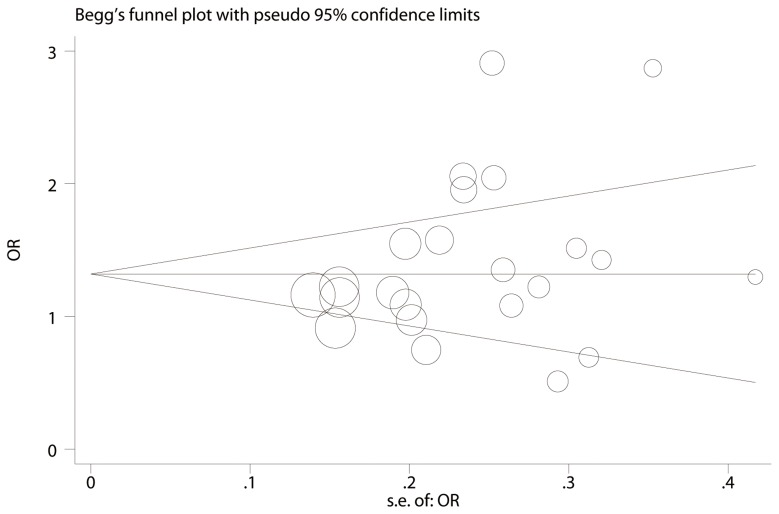
Funnel plot analysis of comparison to detect publication bias in 23 eligible studies. The circles represent the weight of individual study.

Sensitivity analysis was performed to assess the influence of each individual study on the pooled OR by deleting one single study each time, and there was no substantial change in the corresponding pooled OR (Figure S3). Begg’s funnel plot and Egger’s test were performed to assess publication bias. Begg’s funnel plot was roughly symmetrical (p = 0.205, Figure 4). The statistical results still did not show publication bias by Egger’s test (p = 0.115). Therefore, there was no significant publication bias in these eligible studies.

## Discussion

We performed this meta-analysis to explore the association of GSTT1 genotype with lung cancer in Asian population. In this meta-analysis, 23 eligible studies, including 4065 lung cancer cases and 5390 controls, were identified and analyzed. The results suggested that GSTT1 null genotype carriers had an increased risk of lung cancer in Asian population. This meta-analysis was based on all published data and had enough statistical power to detect a modest difference.

At present, many studies have investigated the association between GSTT1 null genotype and cancers, such as oral carcinoma [46], esophageal cancer [47], breast cancer [48], colorectal cancer [49], and pancreatic cancer [50], but the findings were inconsistent, especially in lung cancer. Further, about 20% of Caucasians are homozygous for a GSTT1 null allele. The GSTT1 null genotype is more common in Asians, in with the frequency ranges from 47% to 64% [51]. The GSTs can catalyze the conjugation of the tripeptide glutathione to a wide variety of exogenous and endogenous chemicals with electrophilic functional groups, including products of oxidative stress, environmental pollutants, and carcinogens [12]. GSTT1 null genotype lost this enzyme ability; therefore we speculated that GSTT1 null genotype could increase the risk of lung cancer. However, in 23 eligible studies, only 8 reported positive results, which were in consistent with our pooled analysis. Furthermore, there was one study reported GSTT1 null genotype could decrease the lung cancer risk [40].

In the sub-group analysis of smoking status, there was a significant association in ever-smokers, while no association found in non-smokers. Cigarette smoking is a high risk factor of lung cancer. It may be related to the important role of GSTT1 deletion polymorphism in the metabolism of polycyclic aromatic hydrocarbons contained in cigarette smoking. In addition, in the sub-group of squamous cancer which was strongly associated with smoking, there was no significant association, although 2 individual studies reported GSTT1 null genotype increased lung cancer risk [43], [44]. This discrepancy may be explained by the reason that the sample size of the studies was relatively small. No significant associations were found in the sub-group of adenocarcinoma and small cell carcinoma.

The heterogeneity was not detected by meta-regression. However, through sub-group analyses, we found the heterogeneity might come from source of controls and sample size. When studies were stratified by source of controls and sample size, we conducted heterogeneity test to explore the source of heterogeneity (Table 2). We also found the association between GSTT1 null genotype and lung cancer risk was more significant in the studies of population controls than hospital controls, which indicated that the distribution of GSTT1 null genotype in the hospital control groups might not represent of the general population. The pooled OR was mainly affected by studies of sample size (≤500 participants). Studies of small size may contribute to a small-study effect, in which effects reported are larger, and lead to between studies variance. Therefore, sample size was considered for heterogeneity in this meta-analysis. In addition, the overall number of subgroups such as “sample size >500 participants” and “hospital-based” studies was smaller compared with “sample size ≤500 participants” and “population-based” studies respectively, so it could account for the lack of significant association.

In this meta-analysis, we included 4065 cancer cases and 5390 controls, which can provide enough statistical power and strengthened the reliability of our results. There are some limitations inherent in this meta-analysis. Firstly, individual data was not available and a more precise analysis should be conducted on other covariates such as age, sex, and environmental exposure. Secondly, the number of studies included for sub-group analysis of histological types and smoking status was small. Thirdly, the heterogeneity is difficult to exclude. It may be decided by confounding factors, such as gender, age, genetic diversities, different risk factors in life styles, and the exposure to different environmental factors which are difficult to collect completely.

In summary, this meta-analysis suggests that GSTT1 null genotype is associated with a significantly increased risk of lung cancer in Asian population. To confirm this result, large scale case-control studies with detailed individual information are required.

## Supporting Information

Figure S1 **Forest plot for the association between GSTT1 null genotype and lung cancer risk in Asian population on stratification by source of controls.**
(TIF)

Figure S2 **Forest plot for the association between GSTT1 null genotype and lung cancer risk in Asian population on stratification by** **histological types.** SqCC: squamous cell carcinoma; AC: adenocarcinoma; SCLC: small cell carcinoma.
(TIF)

Figure S3 **Sensitivity Analyses.** The pooled odds ratios were calculated by omitting each data set at a time
(TIF)

Table S1 **PRISMA checklist.**
(DOC)
