# Supplementary figures and images for: GSTT1 Null Genotype Contributes to Lung Cancer Risk in Asian Populations: A Meta-Analysis of 23 Studies
Source: PLoS One. 2013 Apr 24;8(4):e62181. doi: 10.1371/journal.pone.0062181 (PMC3634775; doi:10.1371/journal.pone.0062181)

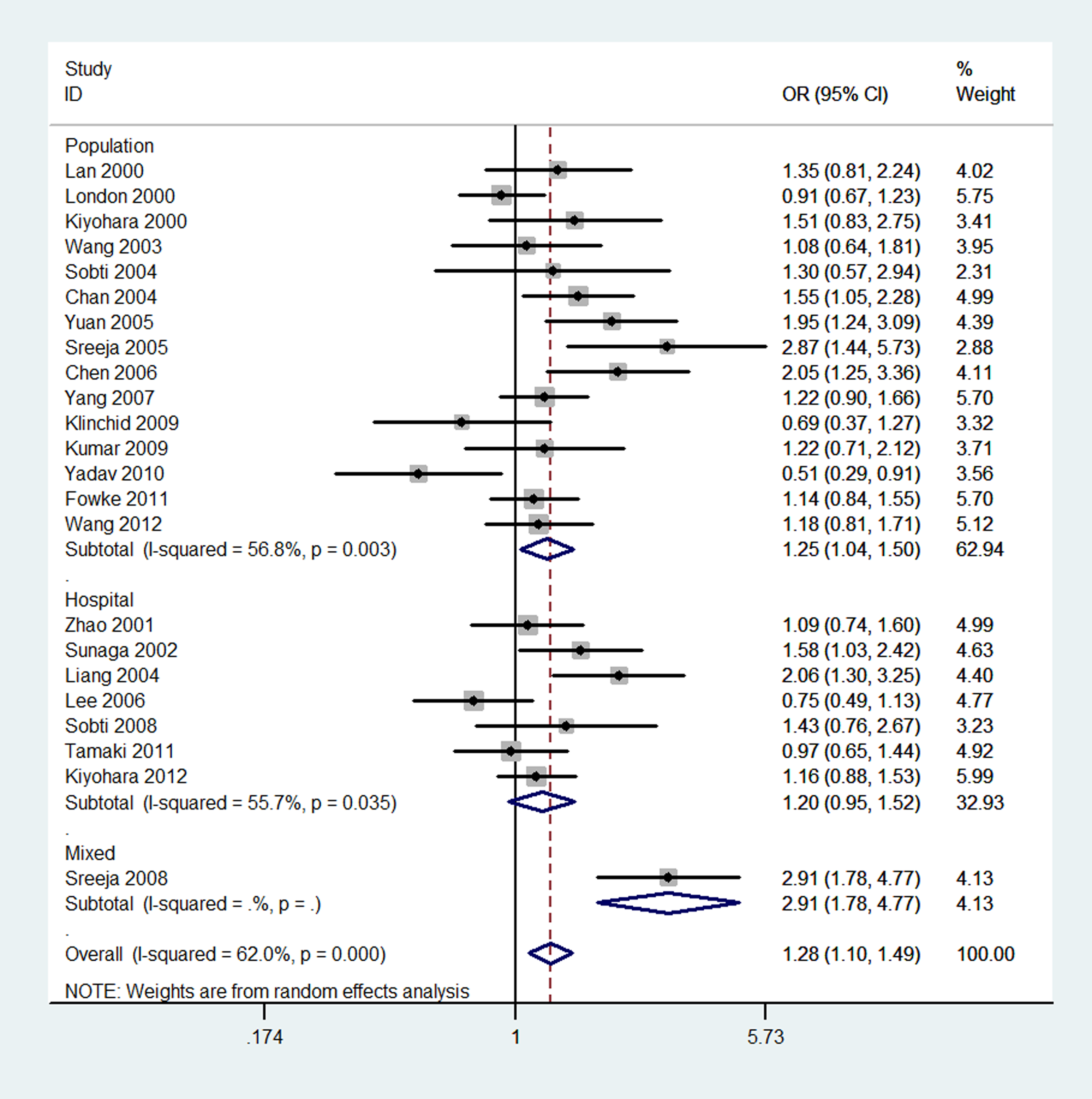

Supplement: Figure S1 — Forest plot for the association between GSTT1 null genotype and lung cancer risk in Asian population on stratification by source of controls. (TIF) [file pone.0062181.s001.tif]

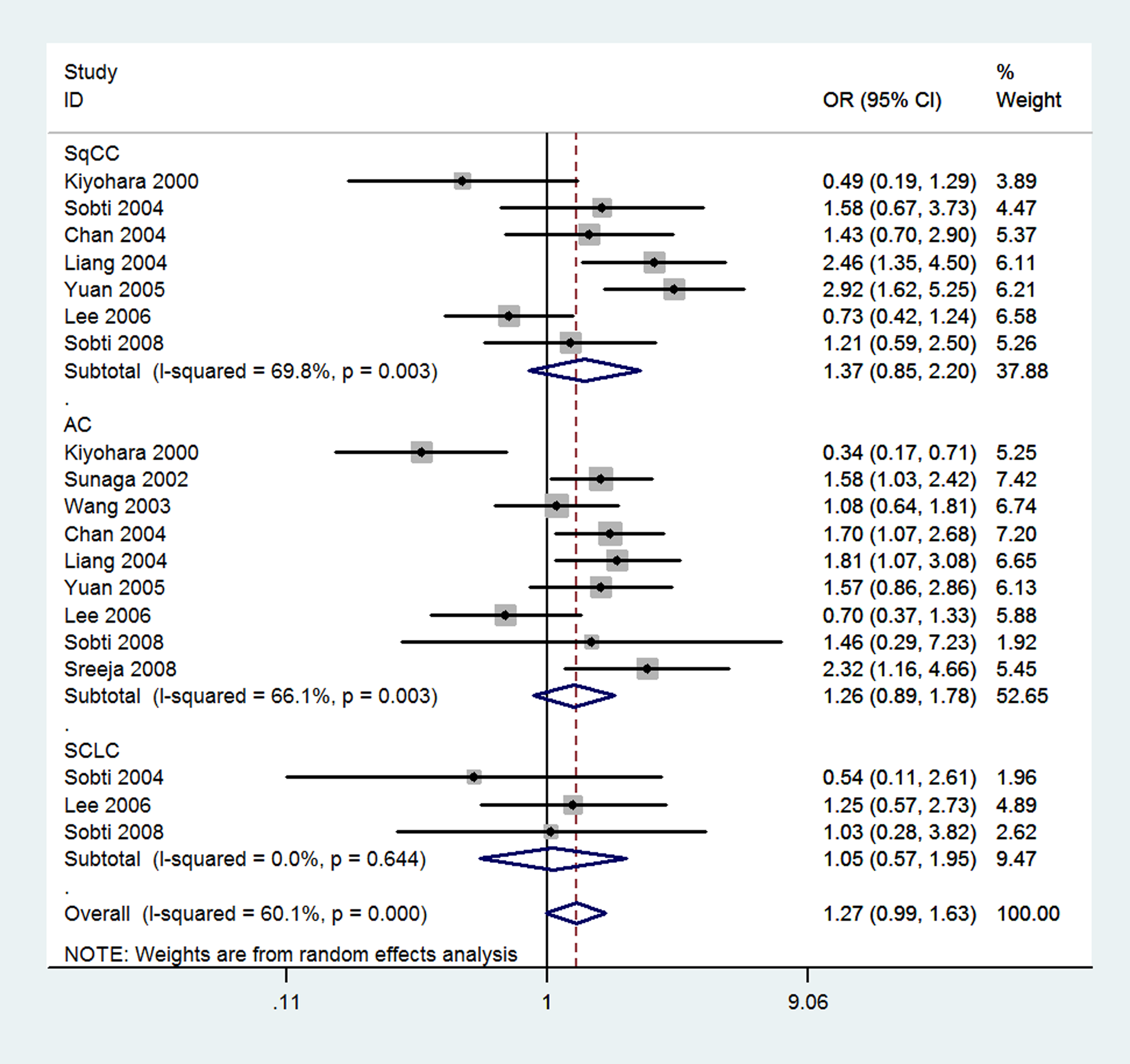

Supplement: Figure S2 — Forest plot for the association between GSTT1 null genotype and lung cancer risk in Asian population on stratification by histological types. SqCC: squamous cell carcinoma; AC: adenocarcinoma; SCLC: small cell carcinoma. (TIF) [file pone.0062181.s002.tif]

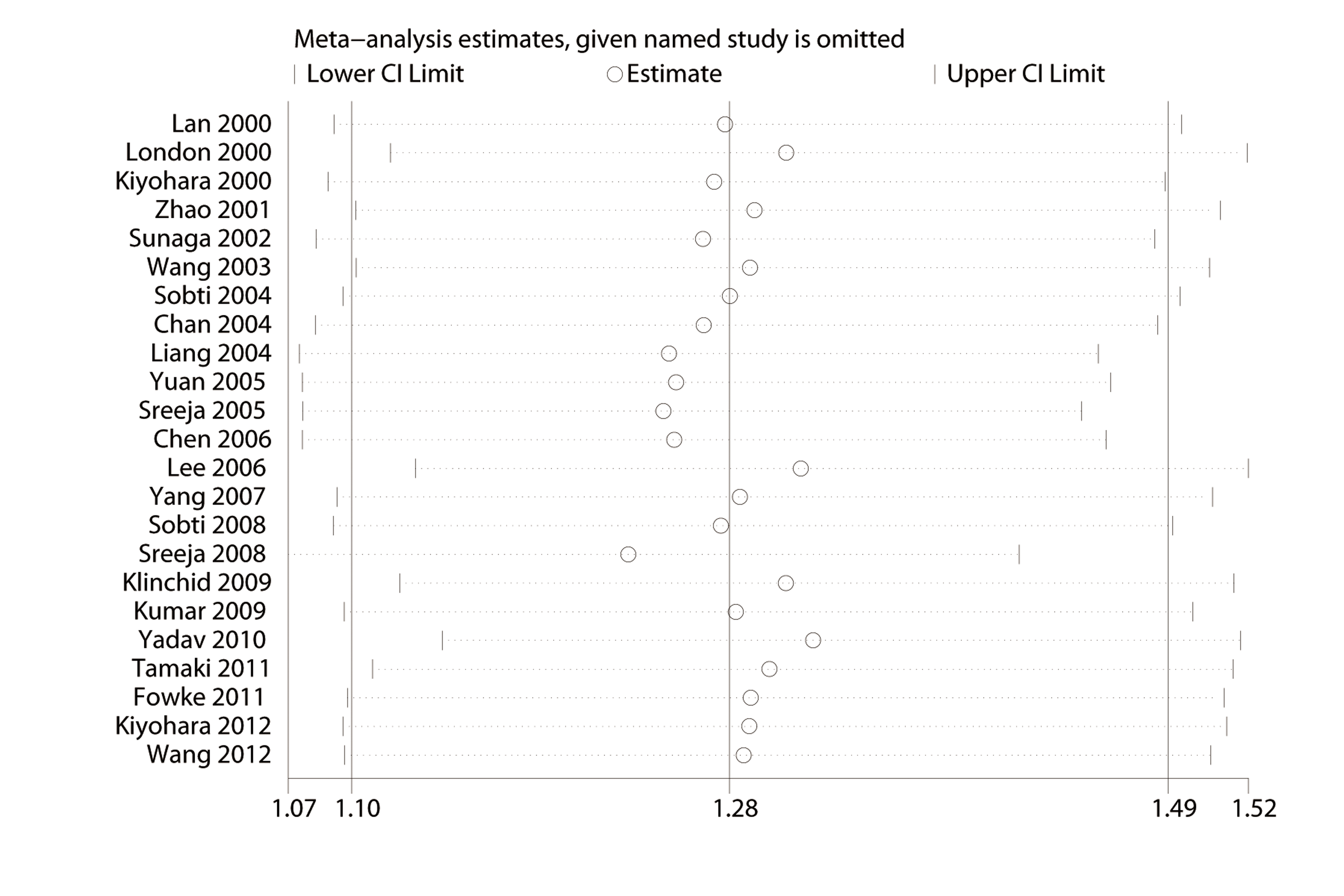

Supplement: Figure S3 — Sensitivity Analyses. The pooled odds ratios were calculated by omitting each data set at a time (TIF) [file pone.0062181.s003.tif]
